# Cost-Effectiveness of Product Reformulation in Response to the Health Star Rating Food Labelling System in Australia

**DOI:** 10.3390/nu10050614

**Published:** 2018-05-14

**Authors:** Ana Maria Mantilla Herrera, Michelle Crino, Holly E. Erskine, Gary Sacks, Jaithri Ananthapavan, Cliona Ni Mhurchu, Yong Yi Lee

**Affiliations:** 1 School of Public Health, Faculty of Medicine, University of Queensland, Brisbane 4001, Australia; holly_erskine@qcmhr.uq.edu.au (H.E.E.); y.lee5@uq.edu.au (Y.Y.L.); 2 Queensland Centre for Mental Health Research (QCMHR), Wacol 4076, Australia; 3 The George Institute for Global Health, University of New South Wales, Sydney 2042, Australia; mcrino@georgeinstitute.org.au (M.C.); c.nimhurchu@auckland.ac.nz (C.N.M.); 4 Institute for Health Metrics and Evaluation, University of Washington, Seattle, WA 98121, USA; 5 Centre for Clinical Research, University of Queensland, Brisbane 4001, Australia; 6 Global Obesity Centre (GLOBE), Centre for Population Health Research, Deakin University, Geelong 3320, Australia; gary.sacks@deakin.edu.au (G.S.); jaithri.ananthapavan@deakin.edu.au (J.A.); 7 Deakin Health Economics, Centre for Population Health Research, Deakin University, Geelong 3320, Australia; 8 National Institute for Health Innovation, University of Auckland, Auckland 1072, New Zealand

**Keywords:** obesity prevention, cost-effectiveness, economic evaluation, Health Star Rating, front-of-pack labelling

## Abstract

The Health Star Rating (HSR) system is a voluntary front-of-pack labelling (FoPL) initiative endorsed by the Australian government in 2014. This study examines the impact of the HSR system on pre-packaged food reformulation measured by changes in energy density between products with and without HSR. The cost-effectiveness of the HSR system was modelled using a proportional multi-state life table Markov model for the 2010 Australian population. We evaluated scenarios in which the HSR system was implemented on a voluntary and mandatory basis (i.e., HSR uptake across 6.7% and 100% of applicable products, respectively). The main outcomes were health-adjusted life years (HALYs), net costs, and incremental cost-effectiveness ratios (ICERs). These were calculated with accompanying 95% uncertainty intervals (95% UI). The model predicted that HSR-attributable reformulation leads to small reductions in mean population energy intake (voluntary: 0.98 kJ/day [95% UI: −1.08 to 2.86]; mandatory: 11.81 kJ/day [95% UI: −11.24 to 36.13]). These are likely to result in reductions in mean body weight (voluntary: 0.01 kg [95% UI: −0.01 to 0.03]; mandatory: 0.11 kg [95% UI: −0.12 to 0.32], and HALYs (voluntary: 4207 HALYs [95% UI: 2438 to 6081]; mandatory: 49,949 HALYs [95% UI: 29,291 to 72,153]). The HSR system evaluated via changes in reformulation could be considered cost-effective relative to a willingness-to-pay threshold of A$50,000 per HALY (voluntary: A$1728 per HALY [95% UI: dominant to 10,445] and mandatory: A$4752 per HALY [95% UI: dominant to 16,236]).

## 1. Introduction

Obesity represents a burden to health and the economy by increasing the risk of non-communicable diseases [1], decreasing health-related quality of life [2], increasing healthcare costs, and decreasing productivity [3,4,5]. In Australia, 63% of adults and 27% of children were classified as overweight or obese (i.e., having a body mass index (BMI) greater than 25 kg/m^2^) [6], with the costs of obesity alone estimated to be A$8.6 billion per year [7]. A primary driver of obesity is the obesogenic (i.e., obesity-promoting) food environment [8]. Recent research has noted the need for initiatives that both improve the energy density of food and help consumers make healthier choices [9,10,11,12]. Front-of-pack labelling (FoPL) is one such initiative that provides consumers with immediate, summary information regarding the nutritional value of a food product at the point of purchase [11,13,14,15,16,17,18]. FoPL involves displaying nutritional information on the front of pre-packaged foods in a clear and uniform manner [17]. FoPL has been frequently recommended as a key component of the policy response to obesity, with examples of FoPL implementation in several countries [19,20,21,22,23]. Evidence suggests that FoPL can help consumers navigate the food environment by providing nutritional information in an easy-to-interpret format which aids healthier food choices [24]. Furthermore, FoPL may serve as an incentive for the food and beverage industry to reformulate their products with a healthier nutritional profile, thus improving their nutritional quality [20,25,26,27,28,29].

The Health Star Rating (HSR) system is a FoPL initiative that was endorsed by both the Australian and New Zealand governments on a voluntary basis in June 2014 [24], with a 5-year implementation review planned to occur in 2019 [30]. The HSR system assesses pre-packaged foods based on their overall nutritional profile and provides a product rating ranging from half a star (least healthy) to five stars (most healthy), displayed as a graphic [24]. A two-year progress review by the Health Star Rating Advisory Committee (HSRAC) showed that 2031 of 14,102 Australian pre-packaged food products had adopted the HSR system by 2016 [30]. Products that utilized the HSR system were more likely to have undergone reformulation compared to products that did not adopt the system, possibly due to manufacturers seeing this as an opportunity to obtain a higher HSR than their competitors [30]. However, the influence of the HSR system on reformulation and any subsequent improvements in population health have not been assessed.

This study examined the long-term health impacts and cost-effectiveness of the reformulation of energy density in Australian pre-packaged foods and beverages attributable to the HSR system. We investigated the impact of the HSR system on obesity by comparing the total energy density of food products before and after the implementation of the HSR system; and estimated subsequent changes in weight and the prevalence of obesity-related diseases across the Australian population. We assessed the impact of the HSR system from a supply-side perspective to estimate how changes in the food environment might impact weight. Furthermore, we evaluated scenarios in which the HSR system is implemented on either a voluntary or mandatory basis.

## 2. Materials and Methods

### 2.1. Analytical Approach

The long-term cost-effectiveness of the HSR system in Australia was evaluated under two scenarios: (1) the current voluntary scheme; and (2) implementation on a mandatory basis. In the voluntary scenario, changes in energy density across an observed uptake (6.7%) of pre-packaged food products that implemented HSR labelling were assumed to be 100% attributable to the HSR system. Data on the energy density of food products included in the analysis were available for the years 2013 (pre-implementation) and in 2016 (post–implementation). The assumption of a change in energy density that was 100% attributable to the HSR-related reformulation was tested in subsequent univariate sensitivity analyses. Estimated changes in energy density were linked to nationally representative consumption of food to obtain changes in average daily energy intake, weighted by Australian consumption data. For the mandatory scenario, we recalculated the magnitude of the intervention effect from the voluntary scenario assuming that 100% of all pre-packaged food products implemented the HSR system. A limited societal perspective was considered appropriate for this evaluation given that the intervention involves a policy approach that impacts population health, the food industry, and the government in the monitoring and promotion of healthier pre-packaged foods in Australia. As such, our analyses include costs to the government and the food industry. This economic evaluation conforms to the Consolidated Health Economic Evaluation Reporting Standards (CHEERS) checklist, as presented in Supplementary Table S1 [31].

### 2.2. Intervention Effect Size

**Figure 1:**
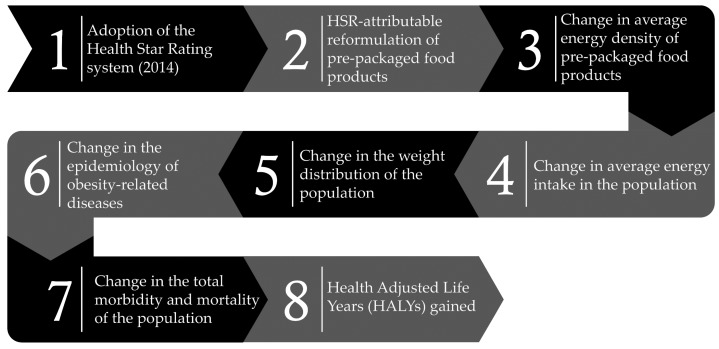
Intervention pathway for the Health Star Rating (HSR) system.

The HSR system was modelled as a national public health intervention that changes the food environment by acting as an incentive for the reformulation of pre-packaged food products purchased by Australian consumers (i.e., a supply-side intervention). Based on the findings of previous studies [32], we assumed that Australians will continue to consume the same quantity of food and beverages despite reductions in energy density of pre-packaged foods (i.e., no compensatory effect on the demand side). This will, in turn, result in changes in the average weight across the population. The effect of HSR-attributable reformulation in both baseline scenarios is determined by extrapolating the observed data, modelling constant effects over time, and assuming that all remaining variables are held constant (ceteris paribus) [33,34,35]. Figure 1 shows the intervention pathway.

Changes in average energy intake due to the HSR system were calculated by using a difference in difference analysis to compare the energy density (kJ per 100 g) of HSR-labelled and non-HSR labelled food products pre- and post-implementation of the HSR system in Australia (2013 and 2016 respectively). This was similar to a previous analysis conducted in New Zealand [29]. The statistical significance of overall changes in energy density resulting from the uptake of the HSR system was evaluated using a paired t-test shown in Supplementary Table S2. We used the Australian FoodSwitch database [36,37], which contains information on Australian pre-packaged food and beverage products available by year [36,37], to obtain product information on average kJ per 100 g and the presence of HSR labelling by food category in 2013 and 2016. Individual products were categorized into 18 food categories according to the Global Food Monitoring Protocol [37]. Food categories with products that were not eligible to carry a HSR label (i.e., alcohol, vitamins and minerals, special foods) or products that were unable to be categorized or reformulated by manufacturers (e.g., eggs) were excluded (*n* = 3008, 17% of products available). After exclusions, 13 food categories were included in the analysis (see Supplementary Table S3). The effect of the intervention was measured at the food category level and assumed to be the result of the HSR intervention in the baseline scenarios.

Changes in the energy density (kJ per 100 g) of labelled and non labelled food products between 2013 and 2016 were converted to changes in kJ per g by food categories to be applied to the Australian food consumption data by food category, age, and sex (i.e., g/day) sourced from the 2011–2012 Australian Health Survey (AHS) [38]. Average changes in daily energy intake (i.e., kJ per day), by age and sex, were calculated using a weighted average change in which changes in the energy density of each food category calculated using the FoodSwitch data, by age and sex, were multiplied by the weight of each food category on the daily energy intake, by age and sex (see Supplementary Tables S4 and S5). Changes in daily energy intake were then transformed into corresponding changes in body weight (measured in kilograms (kg)) using previously derived equations for children and adults [39]. Lastly, average changes in body weight (i.e., kg) were transformed into BMI units using census data on average Australian height, by age and sex, from the 2011–2012 AHS [38]. The likely health impact of the HSR system under a mandatory scheme was estimated using cross-multiplication to estimate changes in daily energy density resulting from a 100% uptake based on the HSR uptake of individual food products’ uptake in the voluntary scenario. The method for calculating the change in energy density resulting from 100% HSR uptake is shown in Equation 1. The mandatory scenario aims to

(1) $$\Delta \;\mathrm{in}\;\mathrm{energy}\;\mathrm{density}{\left(\mathrm{mandatory}\right)}_{a}=\frac{\Delta \;\mathrm{in}\;\mathrm{energy}\;\mathrm{density}{\left(\mathrm{voluntary}\right)}_{a}\times 100\%}{\%\;\mathrm{HSR}\;\mathrm{uptake}{\left(\mathrm{voluntary}\right)}_{a}}$$

where: Δ denotes change; and *a* denotes a specific type of food product.

### 2.3. Health Benefit Modelling

The CRE-Obesity model (see Supplementary Figure S1) [40] was used to estimate the long-term health benefits and costs of population changes in BMI attributable to the HSR system. The CRE-obesity model is a proportional, multi-state, life table Markov model that simulates how intervention-related changes in the population-level distribution of BMI affect the incidence, prevalence and mortality of nine obesity-related diseases, which in turn, affect the overall mortality and morbidity of the modelled population [41]. The long term costs and benefits of the intervention for the 2010 Australian population over the lifetime is modelled by comparing two hypothetical scenarios: (1) an intervention scenario where the BMI distribution for the 2010 Australian population cohort is modified following the implementation of an obesity intervention; and (2) a comparator where the same population cohort is exposed to a ‘do nothing’ scenario, with BMI distributions remaining unchanged in the absence of an intervention. Main outcome variables are incremental health-adjusted life years (HALYs), incremental nets costs, and incremental cost-effectiveness ratios (ICERs) [42]. Incremental HALYs include health-related improvements in morbidity and mortality that occur in the intervention scenario relative to the comparator, while incremental net costs include all intervention costs and cost offsets (i.e., treatment costs averted due to fewer disease cases). ICERs were calculated by dividing incremental net costs by incremental HALYs. This evaluation is part of an obesity-prevention priority-setting study in Australia and uses standardized methods in order for these results to be comparable to other interventions undertaken as part of this study [43,44,45].

### 2.4. Intervention Costs

Intervention costs were obtained from a recent Australian government-commissioned report and are presented in Table 1 [46]. These included costs to industry and the government. Costs to industry are those related to implementation (i.e., costs of redesign, volume, and timeline for changes to product labels) and ongoing costs (i.e., monitoring and additional requirements specific to each product such as change in packing materials). Costs to government are those related to implementation (i.e., education and promotion) as well as ongoing costs of monitoring and evaluating the HSR system. Costs to government were assumed to remain over the lifetime during which the HSR system is in effect. Costs to industry were modelled based on the average length of a labelling cycle, which has been reported to last up to three years [46,47]. Costs for the voluntary scenario were calculated to reflect the 6.7% HSR system uptake observed in 2016 according to FoodSwitch data. For the mandatory scenario, we adjusted costs to reflect 100% HSR system uptake and included the cost of legislating the regulation [43].

Cost offsets (i.e., treatment costs that are averted due to the prevention of obesity-related diseases in the intervention scenario) were calculated using data from the Disease Costing and Impact Study 2000–2001 [48]. The impact of the HSR system on changes in company revenues was not modelled as a previous evaluation found that revenues at industry level were unlikely to change following the uptake of the HSR system [30]. Similarly, costs to individuals were excluded as evidence suggests that a direct increase in costs for consumers is unlikely [49]. All costs were converted to 2010 Australian dollars (2010 A$) using Australian health price deflators from the Australian Institute for Health and Welfare (AIHW) [50].

### 2.5. Cost-Effectiveness Modelling

The intervention was considered cost-effective if resulting ICERs were below an Australian willingness-to-pay (WTP) threshold of A$50,000 per HALY [51]. All costs and benefits were measured over a lifetime horizon (up to 100 years or death) and were discounted at a 3% annual rate.

Uncertainty distributions were assigned to input parameters to account for the impact of sampling uncertainty (i.e., standard errors) on the final cost-effectiveness results [52]. Uncertainty distributions were based on known properties of input parameters [53]. Table 2 presents a summary of input parameters with uncertainty distributions, assumptions and sources. The Ersatz program (version 1.31, Sunrise Beach, Australia; available at: http://www.epigear.com) was used to perform Monte Carlo simulation based on 2000 uncertainty iterations. Main model outcomes are presented as HALYs, net costs, and ICERs with 95% uncertainty intervals (95% UI) [54]. The 2000 uncertainty iterations produced by the Monte Carlo simulation were graphically illustrated on cost-effectiveness (CE) planes with incremental benefits plotted on the horizontal axis and incremental costs plotted on the vertical axis [55]. The CE plane is a scatterplot divided into four quadrants that graphically denotes how the costs and benefits arising from an intervention compare to those of the comparator. For example, uncertainty iterations that lie on the South-East (SE) quadrant signify that the intervention is less costly and more effective than the comparator (i.e., the intervention is ‘dominant’). Conversely, uncertainty iterations in the North-West (NW) quadrant signify that the intervention is more costly and less effective than the comparator (i.e., the intervention is ‘dominated’). Lastly, uncertainty iterations in the North-East (NE) quadrant signify an intervention that is more costly and more effective than the comparator. In this case, the cost-effectiveness of the intervention is usually determined by whether uncertainty iterations lie below a particular WTP threshold [55].

The baseline analysis assumed that 100% of observed changes in energy density before and after the HSR implementation were attributable to the intervention (i.e., 100% HSR-attributable energy reformulation). However, we deemed this assumption too heroic and tested the impact of lower proportions of HSR-attributable energy intake (i.e., 50%, 30% and 10%) in univariate sensitivity analyses under the voluntary scenario.

## 3. Results

### 3.1. Intervention Effect Size

Among pre-packaged food products available in both 2013 and 2016 (*n* = 14,986), 6.7% (95% UI: 6.3% to 7.1%) implemented the HSR system. When comparing pre-packaged food products across both years, we found that those with a HSR label led to greater reductions in energy density than food products without a HSR label (see Table 3). Small but statistically significant reductions in energy density were found in the 1004 food products that displayed a HSR label in 2016 (−7.11 kJ/100g 95% UI: −14.2 to −0.1, *p* value = 0.04). Changes in energy density by food categories are presented in Supplementary Table S6. Overall, a comparison between the energy density of HSR and non-HSR labelled food products available in 2013 and 2016 showed an average reduction in daily energy intake of 0.98 kJ per day (95% UI: −1.08 to 2.86), which led to an average weight reduction of 0.01 kg (95% UI: −0.03 to 0.01) and an average BMI reduction of 0.003 kg/m^2^ (95% UI: −0.009 to 0.003). Model inputs of changes in energy density and their corresponding weight reduction in kg and BMI are presented, by age and sex, in Supplementary Tables S7 and S8. Increasing the coverage of the HSR system to all products available in 2016 (i.e., the mandatory scenario) resulted in average daily energy intake reductions of 11.81 kJ per day (95% UI: −11.24 to 36.13), which led to an average weight reduction of 0.11 (95% UI: −0.12 to 0.32) kg and an average BMI reduction of 0.04 kg/m^2^ (95% UI: −0.040 to 0.115).

### 3.2. Cost-Effectiveness Modelling

**Figure 2:**
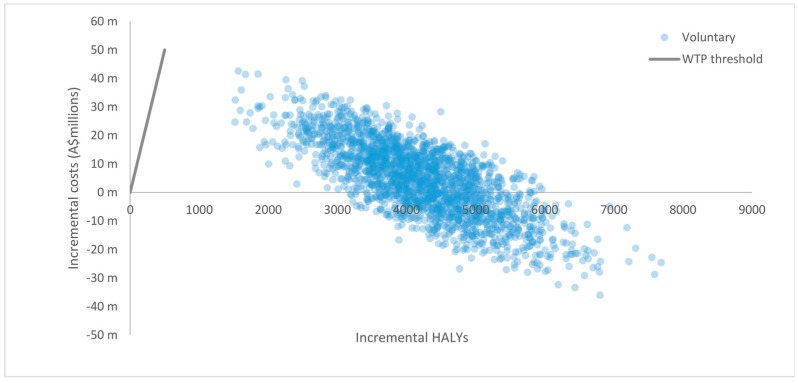
Cost-effectiveness plane for the HSR system under the voluntary scenario (6.7% uptake).

**Figure 3:**
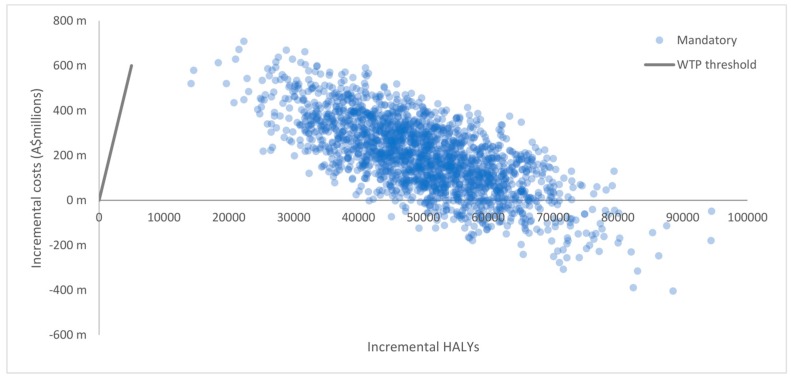
Cost-effectiveness plane for the HSR system under the mandatory scenario (100% uptake).

**Figure 4:**
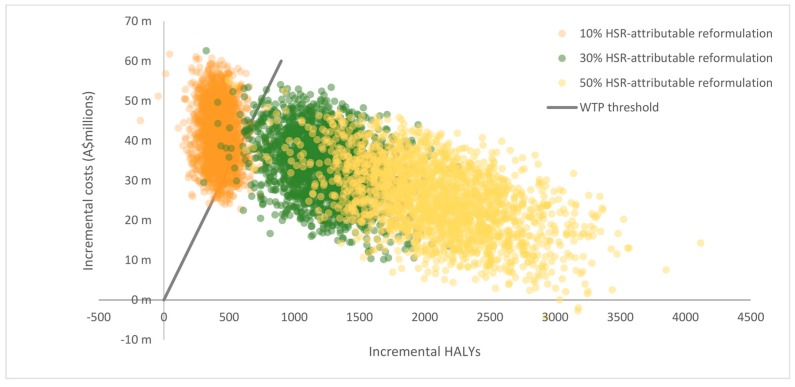
Cost-effectiveness plane for sensitivity analyses of the HSR-attributable reformulation under the voluntary scenario.

Results from baseline scenarios are presented in Table 4. The model predicted that the voluntary implementation of the HSR system was cost-effective (A$1728 per HALY, 95% UI: dominant to 10,446) assuming a WTP threshold of A$50,000 per HALY over the lifetime of the 2010 Australian population. We found that the mandatory implementation of the HSR system was also cost-effective (A$4752 per HALY, 95% UI: dominant to 16,236). The HSR system was found to be cost-effective under both baseline scenarios with uncertainty iterations that spanned between the SE quadrant and the area below the WTP threshold in the NE quadrant, which signifies that the intervention is cost saving with respect to the ‘do nothing’ scenario. Graphical illustrations of results are presented in CE planes shown in Figure 2 and Figure 3. As shown in Figure 2, the majority of uncertainty iterations for the voluntary scenario were mostly situated around the NE quadrant (64%) and SE quadrant (36%), meaning that despite the costs of the intervention, it has larger benefits compared to the ‘do nothing’ scenario. Similarly, Figure 3 shows that the majority of uncertainty iterations for the mandatory scenario were also situated in the NE quadrant (89%) and SE quadrant (11%). Overall, results for both the voluntary and mandatory scenarios remained well below the WTP threshold in the NE quadrant, highlighting the cost-effectiveness of the HSR. The level of HSR uptake was a key driver for the magnitude of intervention impacts and the uncertainty around estimates. As such, incremental HALYs and costs saved in the mandatory scenario exceeded those in the voluntary scenario. Results from sensitivity analyses indicate that the effectiveness of the voluntary implementation of the HSR system is sensitive to the proportion of HSR-attributable reformulation as shown in Table 5. The voluntary HSR system remained cost-effective when the assumed level of HSR-attributable reformulation was reduced from 50% to 30% (A$13,374 per HALY [95% UI: 3044 to 31,940] and A$29,006 per HALY [95% UI: 11,428 to 59,863], respectively). However, when the level of HSR-attributable reformulation was reduced to 10% the intervention was not cost effective (A$106,368 per HALY [95% UI: 54,072 to 191,145]). As shown in Figure 4, all iterations in the sensitivity analysis are situated in the NE quadrant (100%), with results closer and situated to the left of the cost-effectiveness threshold, meaning that if the HSR has a lower impact on reformulation, the intervention would not be cost-effective.

## 4. Discussion

The findings of this study indicate that, from a limited societal perspective, the HSR system’s impact on product reformulation has the potential to generate increasing health benefits and reduce mean population body weight, with the HSR system found to be cost-effective under the voluntary and mandatory scenarios relative to a willingness-to-pay threshold of A$50,000 per HALY (A$1728 per HALY [95% UI: dominant to 10,445] and A$4752 per HALY [95% UI: dominant to 16,236], respectively). This study found that the average energy density of food products that implemented the HSR system was lower post-HSR (e.g., average energy density of non-alcoholic beverages was 17,516 kJ per 100 g in 2013 compared with 14,320 kJ per 100 g in 2016). We attributed this change to the implementation of the HSR system, based on the observation that the reductions in energy density in products with HSR were greater than for similar products that did not implement the HSR system. Specific examples illustrate the effect at a product level. Prior to the introduction of HSR, the energy density of ‘thin corn cakes’ in 2013 was 2031 kJ per 100 g, and, at that stage, the product would have received 2 stars. Post-implementation of the HSR system on that product (in 2016), the energy density was 1680 kJ per 100 g, and the product displayed 3 stars. On the contrary, products that did not implement the HSR showed lower or even positive increases in energy density. For instance, the energy density of ‘Fruit and Muesli’ in 2013 (pre HSR implementation) was 919 kJ per 100 g and the energy density of the same product in 2016 was 1110 kJ per 100 g. On average, the energy density of food products available in 2013 that implemented the HSR system in 2016 was found to be lower (*p* < 0.05) than products that did not implement the HSR system. The implementation of the HSR is likely to have incentivized energy density reductions of food products to elicit higher HSR ratings (e.g., ‘Gluten free pizza base’ was 1400 kJ per 100 g in 2013 and 1015 kJ per 100 g in 2016; this change assigns the product 3.5 stars, rather than the 3 stars it originally would have obtained). While these reductions in daily energy density result in small reductions in average population energy intake (0.98 kJ per day, 95% UI −1.08 to 2.86) and average body weight (0.01 kg 95% UI −0.01 to 0.03), the long-term health impacts are nevertheless likely to be substantial as shown in Table 4 and Table 5. Long-term health impacts are the result of relatively low intervention costs (A$46.1 m [95% UI: 32.0 m to 59.7 m]) and lifetime health benefits accrued from an intervention that modifies the food environment.

Findings from this study, which calculated health benefits attributable to the HSR system from supply-side changes (i.e., not including the potential benefits of changes in behavior on the demand side), suggest that small changes in the average energy intake of the population can potentially reduce population levels of obesity and, in turn, the burden of obesity-related diseases. An earlier cost-effectiveness study found that another FoPL intervention implemented in Australia (i.e., an intervention involving “traffic-light” nutrition labelling on food products such that green, amber and red colors denote the comparative nutritional value of different products) was cost-saving, with a mean population weight reduction of 1.3 kg and incremental health benefits equating to 45,100 HALYs gained (95% UI: 37,700 to 60,100) [59]. This study only included four food categories (i.e., breakfast cereals, pastries, mixed dishes with cereal, and sausages), used 2003 as baseline year, and used different assumptions in the estimation of intervention effects (i.e., the intervention applied across a 10-year time horizon and impacted on 2.5% of the adult population). While these methodological differences limit the direct comparability between our study and the previous study, both studies nonetheless demonstrate that front-of-pack nutritional labels are likely to represent ‘value for money’ when intervening to reduce the population burden of obesity in Australia. Findings from our study are also in line with a recent cost-effectiveness study on energy density reductions resulting from reformulation of sugar-sweetened beverages (SSBs) in Australia [60]. Reformulation of SSBs was found to be cost saving, with substantial increases in health benefits if the intervention was implemented across all products [60].

However, findings from this study also indicate that modelling results should be interpreted with caution. As shown in our sensitivity analysis, the proportion of HSR-attributable reformulation modified the effectiveness of the intervention, making the ‘do-nothing’ scenario better than the intervention if the HSR only contributes to 10% of energy density reformulation. Food labelling has been found to promote product reformulation, which in turn leads to improvements in the energy density of food [22,26,27,61]. Nevertheless, there is no clear evidence on the magnitude of the effect of HSR on reformulation. Furthermore, in this analysis we have assumed that consumers did not change their purchasing or eating behaviors in response to changes in the energy density of food products. Our assumption that no compensatory eating or purchasing behavior would occur in response to reductions in energy density of packaged foods might have overestimated intervention impacts. However, we believe it is more likely that we have underestimated the impact of the HSR on energy intake as the HSR may help consumers choose healthier options, which we were unable to fully capture with the data available. Given the absence of data on HSR-attributable reformulation and the assumption of no changes in consumers’ behavior, we applied wide uncertainty intervals around the intervention effect size to capture a greater margin of error (i.e., we assumed that the standard deviation of the overall intervention effect on weight was equal to its mean) [62].

Moreover, we only accounted for trends in product reformulation in a limited way by conducting a difference-in-difference analysis before and after the implementation of the HSR system. Thus, it is possible that manufacturers who implemented the voluntary system were those with products that were more conducive to reformulation and that this may have occurred independent of the HSR system being implemented. This will impact the attribution of effects to the HSR system in this study; particularly among products that are not conducive to reformulation or reformulation due to other factors. Nonetheless, as shown in our sensitivity analysis even if the HSR only accounts for 30% of product reformulation, the change in the food environment will likely result in health benefits. Similarly, there is a likelihood of incremental changes to the energy density of food products conducted throughout several years, rather than as a once off. In this case, health impacts over time would likely increase as a result of incremental benefits instead of constant benefits over time. This study has also limitations as result of the modelling methodology used. The use of a cohort-population model relies on homogeneity assumptions within age-sex group cohorts which are likely to distort reality by over/under estimating the effect on individuals. Furthermore, the intervention was evaluated as if implemented over the lifetime, based on its capacity to modify the environment, but acknowledges that if following interventions find a significant drop in effectiveness at a particular point in time, the interventions may not be as cost-effective. It is also plausible that there is a time lag between the implementation of the HSR and likely changes in the energy density of food products, which would lead to a current overestimation of ICERs 

Notwithstanding this, there are additional impacts on product reformulation beyond changes in energy density that have not been accounted for in this analysis, which might result in increased beneficial health impacts over time. A two-year progress review of the HSR system noted several nutritional improvements in addition to reductions in energy density [24]. These included reductions in sugars, saturated fat, and sodium, all of which, independent of weight changes, may have an impact on some chronic diseases such as cardiovascular disease and diabetes. Similarly, we modelled intervention costs to the government over the lifetime, unlike the HSR report which modelled these costs over 3 years [24], likely overestimating the intervention costs and thus underestimating the intervention cost-effectiveness. This analysis illustrates that the HSR intervention is likely to be cost-effective to the health sector and society, inducing changes in the BMI profile of the Australian population and consequently leading to substantial improvements in the health of the population. This economic evaluation highlights the potential for FoPL interventions to lead to positive population health outcomes in a cost-efficient manner.

The HSR system has been criticized by some stakeholders for having some anomalies in classifying the healthiness of foods (e.g., some energy-dense foods that are deemed unnecessary for a healthy diet and are classified as ‘discretionary’ or ‘optional’ according to the Australian Dietary Guidelines receive a relatively high HSR rating) [63]. However, the Australian government is reviewing the HSR algorithm and other aspects of the system, with a view to improving the way in which it operates [64]. This analysis has shown that, even in its current form, implementation of the HSR system may still lead to positive overall changes in product reformulation. Our estimates of the cost-effectiveness of the HSR system were limited by data availability and will be improved as evidence from the five-year HSR system review, due in 2019, is obtained, providing a stronger evidence-base to inform policymaking.

## 5. Conclusions

This economic evaluation found that the HSR system, evaluated via energy density reformulation, is likely to be cost-effective, assuming at least 11% of HSR-attributable reformulation. While costs and benefits of the HSR system beyond changes in energy density due to reformulation should be considered, this study provides evidence-based information that can assist policymakers to make informed decisions about future FoPL initiatives targeting obesity. Policy-driven approaches like the HSR system are an important complement to other policy approaches already in place to target obesity (e.g., education campaigns) as they modify the food environment.

## Figures and Tables

**Table 1:** Summary of intervention costs associated with the HSR system.

Costing Items	Value in 2010 A$	
	Voluntary	Mandatory
1. Cost to industry ^a^	2.5 m (1.2 m, 3.7 m)	37.0 m (18.5 m, 55.4 m)
2. Cost to government ^b^	1.2 m (0.6 m, 1.8 m)	17.8 m (8.9 m, 26.7 m)
2.1 Cost of legislation ^c^	not applicable	1.1 m (1.0 m, 1.2 m)

^a^ Cost to the food industry related to costs of labelling and packing changes, such as re-design, labour, overhead and implementation costs. ^b^ Cost to the government related to administrative costs, education campaigns, enforcement, oversight and promotion of the HSR system. ^c^ Only used for mandatory scenario. Abbreviations: A$: Australian dollars; m: millions.

**Table 2:** Input parameters, uncertainty distributions and sources.

Input Parameters	Uncertainty Distribution	Assumptions	Data Sources
Change in weight resulting from the intervention	Normal	The point estimate was the mean obtained from the data source. A standard deviation was assigned to this point estimate that was equal to the mean in the absence of relevant data.	Based on data from the Food Switch database and the Australian Health survey
Intervention costs to industry	Pert	The point estimate (obtained from the data source) was assigned a range of likely minimum and maximum values based on expert opinion.	Based on the projected costs from the HSR report [24]
Intervention costs to government	Pert	The point estimate (obtained from the data source) was assigned a range of likely minimum and maximum values based on expert opinion [53,56].	Based on the projected costs from the HSR report [24]. Costs to the government related to passing a legislation were only applied to the mandatory scenario and were modelled using a gamma distribution [43]
2010 Australian population BMI by age and sex	Lognormal	The mean and standard deviation for each population cohort (by age and sex) was obtained from the data source. A lognormal distribution was used to: restrict the occurrence of values between the interval [0, +∞]; and account for the positively skewed BMI distribution observed in the population [57].	Sourced from the Australian Bureau of Statistics [38]
Relative risks of obesity-related diseases per 5-unit increase of BMI	Lognormal	The mean was obtained from the data source and the standard deviation was calculated as the lognormal of the mean. A lognormal distribution was used to restrict the occurrence of values between [0, +∞].	Sourced from the Global Burden of Disease study [58]

Abbreviations: BMI: body mass index.

**Table 3:** Comparison of food products with and without the HSR label between 2013 and 2016.

Food Category	Average Energy Density (kJ per 100 g) in 2013		Average Energy Density (kJ per 100 g) in 2016		Change in Average Energy Density between 2013 and 2016		% Change in kJ per 100 g (from Baseline)		% Change in kJ per 100 g Attributable to HSR
	With HSR	Without HSR	With HSR	Without HSR	With HSR	Without HSR	With HSR	Without HSR	
Bread and bakery products	1585	1588	1581	1586	−3.3	−2.2	−0.2	−0.1	−0.1
Cereal and grain products	1521	1370	1513	1360	−7.9	−10.4	−0.5	−0.8	0.2
Confectionery	2070	1720	2089	1724	19.7	4.0	1.0	0.2	0.7
Convenience foods	444	512	433	509	−10.9	−3.2	−2.5	−0.6	−1.8
Dairy	608	933	594	932	−13.4	−0.9	−2.2	−0.1	−2.1
Edible oils and oil emulsions	2724	3066	2706	3071	−18.1	5.0	−0.7	0.2	−0.8
Fish and fish products	721	693	720	693	−1.0	0.0	−0.1	0.0	−0.1
Fruit and vegetables	881	998	881	999	−0.6	0.6	−0.1	0.1	−0.1
Meat and meat products	828	878	824	882	−4.1	3.9	−0.5	0.4	−0.9
Non-alcoholic beverages	213	197	208	195	−4.6	−2.1	−2.1	−1.1	−1.1
Sauces, dressings, spreads and dips	1046	816	981	810	−64.7	−5.5	−6.2	−0.7	−5.5
Snack foods	2013	1883	2079	1882	65.8	−0.8	3.3	0.0	3.3
Sugars, honey and related products	1454	1404	1435	1406	−19.7	1.6	−1.4	0.1	−1.5

**Table 4:** Cost-effectiveness results baseline scenarios.

Outputs	Voluntary Scenario * (6.7% HSR Uptake)	Mandatory Scenario * (100% HSR Uptake)
Incremental intervention costs (95% UI) in 2010 A$ millions	46.1 m (32.0 m to 60.2 m)	686.4 m (483.5 m to 894.9 m)
Cost offsets ** (95% UI) in 2010 A$ millions	−41.6 m (−61.6 m to −22.1 m)	−488.7 m (−722.8 m to −265.9 m)
Net incremental costs (95% UI) in 2010 A$ millions	4.5 m (−21.2 m to 28.2 m)	197.7 m (−123.2 m to 513.3 m)
Incremental HALYs (95% UI)	4207 (2438 to 6081)	49,949 (29,291 to 72,153)
Mean ICER (95% UI) in 2010 A$ per HALY	1728 (dominant to 10,445)	4752 (dominant to 16,236)

* Assuming 100% HSR-attributable reformulation. ** Negative values are cost savings. Abbreviations: A$: Australian dollars; HALY: health-adjusted life years; HSR: Health Star Rating; m: million; ICER: incremental cost-effectiveness ratio; UI: uncertainty intervals.

**Table 5:** Sensitivity analyses on HSR-attributable reformulation from the voluntary scenario results.

Outputs	50% HSR-Attributable Reformulation	30% HSR-Attributable Reformulation	10% HSR-Attributable Reformulation
Incremental intervention costs (95% UI) in A$ millions	46.1 m (32.0 m to 59.7 m)	46.0 m (31.7 m to 59.9 m)	46.0 m (32.4 m to 60.0 m)
Cost offsets (95% UI) in A$ millions	−20.9 m (−31.4 m to −11.3 m)	−12.5 m (−18.3 m to −6.5 m)	−4.2 m (−6.3 m to −2.2 m)
Net incremental costs (95% UI) in A$ millions	25.3 m (7.9 m to 42.4 m)	33.5 m (18.0m to 48.5 m)	41.8 m (28.2 m to 56.2 m)
Incremental HALYs (95% UI)	2101 (1226 to 3116)	1253 (702 to 1840)	424 (235 to 618)
Mean ICER (95% UI) A$ per HALY	13,374 (3044 to 31,940)	29,006 (11,427 to 59,863)	106,368 (54,072 to 191,145)

Abbreviations: A$: Australian dollars; HALY: health-adjusted life years; HSR: Health Star Rating; ICER: incremental cost-effectiveness ratio; m: million; UI: uncertainty intervals.

## Supplementary Materials

The following are available online at http://www.mdpi.com/2072-6643/10/5/614/s1, Figure S1: Schematic overview of the CRE-Obesity model, Table S1: Consolidated Health Economic Evaluation Reporting Standards (CHEERS) checklist, Table S2: Paired two sample t test of changes in energy density attributable to the implementation of the HSR between HSR labelled products in 2013 and 2016, Table S3: Total number of food products included by food categories and use of HSR labelling, Table S4: Consumption weights of food categories on total daily energy intake for males, Table S5: Consumption weights of food categories on total daily energy intake for females, Table S6: Composition of food products displaying HSR in 2016 and composition of the same products in 2013, Table S7: Intervention effect size for males by age, Table S8: Intervention effect size for females by age.
