# Erratum: Cost-Effectiveness of Product Reformulation in Response to the Health Star Rating Food Labelling System in Australia; *Nutrients* 2018, *10*, 614

**DOI:** 10.3390/nu10081037

**Published:** 2018-08-08

**Authors:** Ana Maria Mantilla Herrera, Michelle Crino, Holly E. Erskine, Gary Sacks, Jaithri Ananthapavan, Cliona Ni Mhurchu, Yong Yi Lee

**Affiliations:** 1 School of Public Health, Faculty of Medicine, University of Queensland, Brisbane 4001, Australia; holly_erskine@qcmhr.uq.edu.au (H.E.E.); y.lee5@uq.edu.au (Y.Y.L.); 2 Queensland Centre for Mental Health Research (QCMHR), Wacol 4076, Australia; 3 The George Institute for Global Health, University of New South Wales, Sydney 2042, Australia; mcrino@georgeinstitute.org.au (M.C.); c.nimhurchu@auckland.ac.nz (C.N.M.); 4 Institute for Health Metrics and Evaluation, University of Washington, Seattle 98121, WA, USA; 5 Centre for Clinical Research, University of Queensland, Brisbane 4001, Australia; 6 Global Obesity Centre (GLOBE), Centre for Population Health Research, Deakin University, Geelong 3320, Australia; gary.sacks@deakin.edu.au; 7 Deakin Health Economics, Centre for Population Health Research, Deakin University, Geelong 3320, Australia; jaithri.ananthapavan@deakin.edu.au; 8 National Institute for Health Innovation, University of Auckland, Auckland 1072, New Zealand

The authors have requested that the following changes be made to their paper [1].

## Correction 1

The following content in the Abstract on Page 1:

“The model predicted that HSR-attributable reformulation leads to small reductions in mean population energy intake (voluntary: 0.98 kJ/day [95% UI: −1.08 to 2.86]; mandatory: 11.81 kJ/day [95% UI: −11.24 to 36.13]). These are likely to result in reductions in mean body weight (voluntary: 0.01 kg [95% UI: −0.01 to 0.03]; mandatory: 0.11 kg [95% UI: −0.12 to 0.32], and HALYs (voluntary: 4207 HALYs [95% UI: 2438 to 6081]; mandatory: 49,949 HALYs [95% UI: 29,291 to 72,153]). The HSR system evaluated via changes in reformulation could be considered cost-effective relative to a willingness-to-pay threshold of A$50,000 per HALY (voluntary: A$1728 per HALY [95% UI: dominant to 10,445] and mandatory: A$4752 per HALY [95% UI: dominant to 16,236]).”

Was changed to:

“The model predicted that HSR-attributable reformulation leads to small changes in mean population energy intake (voluntary: −0.98 kJ/day; mandatory: −11.81 kJ/day). These are likely to result in changes in mean body weight (voluntary: −0.01 kg [95% UI: −0.012 to −0.006]; mandatory: −0.11 kg [95% UI: −0.14 to −0.07]), and HALYs gained (voluntary: 4207 HALYs gained [95% UI: 2438 to 6081]; mandatory: 49,949 HALYs gained [95% UI: 29,291 to 72,153]). The HSR system could be considered cost-effective relative to a willingness-to-pay threshold of A$50,000 per HALY (incremental cost effectiveness ratio for voluntary: A$1728 per HALY [95% UI: dominant to 10,445] and mandatory: A$4752 per HALY [95% UI: dominant to 16,236])”

In Results, Section 3.1 on page 6, line 7 to line 15:

“Overall, a comparison between the energy density of HSR and non-HSR labelled food products available in 2013 and 2016 showed an average reduction in daily energy intake of −0.98 kJ per day (95% UI: −1.08 to 2.86), which led to an average weight reduction of 0.01 kg (95% UI: −0.03 to 0.01) and an average BMI reduction of 0.003 kg/m^2^ (95% UI: −0.009 to 0.003). Model inputs of changes in energy density and their corresponding weight reduction in kg and BMI are presented, by age and sex, in Supplementary Tables S7 and S8. Increasing the coverage of the HSR system to all products available in 2016 (i.e., the mandatory scenario) resulted in average daily energy intake reductions of 11.81 kJ per day (95% UI: −11.24 to 36.13), which led to an average weight reduction of 0.11 (95% UI: −0.12 to 0.32) kg and an average BMI reduction of 0.04 kg/m^2^ (95% UI: −0.040 to 0.115).”

Was changed to:

“Overall, a comparison between the energy density of HSR and non-HSR labelled food products available in 2013 and 2016 showed an average change in daily energy intake of −0.98 kJ per day, which led to an average weight change of −0.01 kg (95% UI: −0.012 to −0.006) and an average BMI change of −0.003 kg/m^2^ (95% UI: −0.004 to −0.002). Model inputs of changes in energy density and their corresponding weight change in kg and BMI are presented, by age and sex, in Supplementary Tables S7 and S8. Increasing the coverage of the HSR system to all products available in 2016 (i.e., the mandatory scenario) resulted in average daily energy intake change of −11.81 kJ per day, which led to an average weight change of −0.11 kg (95% UI: −0.14 to −0.07) and an average BMI change of −0.04 kg/m^2^ (95% UI: −0.05 to −0.03).”

In Discussion, Page 11, lines 22 to line 24:

“While these reductions in daily energy density result in small reductions in average population energy intake (0.98 kJ per day, 95% UI −1.08 to 2.86) and average body weight (0.01 kg 95% UI −0.01 to 0.03), the long-term health impacts are nevertheless likely to be substantial as shown in Tables 4 and 5.”

Was changed to:

“While these reductions in energy density result in small changes in average population energy intake (−0.98 kJ per day) and average body weight (−0.01 kg, 95% UI: −0.012 to −0.006), the long-term health impacts are nevertheless likely to be substantial as shown in Tables 4 and 5.”

## Correction 2

In Results, Section 3.2 on page 8, lines 5 to line 8:

“The HSR system was found to be cost-effective under both baseline scenarios with uncertainty iterations that spanned between the SE quadrant and the area below the WTP threshold in the NE quadrant, which signifies that the intervention is cost saving with respect to the ‘do nothing’ scenario.”

Was changed to:

“The HSR system was found to be cost-effective when compared to a ‘do nothing’ comparator across both scenarios, with uncertainty iterations that spanned between the SE quadrant (which signifies that the intervention is cost saving) and the area below the WTP threshold in the NE quadrant (which signifies that the intervention is cost-effective).”

The authors apologize for any inconvenience caused to the readers by the changes, stating it does not affect the scientific results. The original manuscript will remain online on the article webpage, with a reference to this Erratum.
