# Supplementary material for: Cost-Effectiveness of Product Reformulation in Response to the Health Star Rating Food Labelling System in Australia
Source: Nutrients. 2018 May 14;10(5):614. doi: 10.3390/nu10050614 (PMC5986494; doi:10.3390/nu10050614)
Supplement: Supplementary file 1 [file nutrients-10-00614-s001.zip › nutrients-295108-sup.docx]

Online supplementary materials

Supplementary Table 1.

| Section/item | Item No | Recommendation | Reported on page No/ line No |
| --- | --- | --- | --- |
| Title and abstract | | | |
| Title | 1 | Identify the study as an economic evaluation or use more specific terms such as “cost-effectiveness analysis”, and describe the interventions compared. | Page 1, lines 2 to 4 |
| Abstract | 2 | Provide a structured summary of objectives, perspective, setting, methods (including study design and inputs), results (including base case and uncertainty analyses), and conclusions. | Page 1, lines 20 to 34 |
|  | | | |
| Background and objectives | 3 | Provide an explicit statement of the broader context for the study. | Page 2, lines 40 to 55 |
|  |  | Present the study question and its relevance for health policy or practice decisions. | Page 2, lines 67 to 74 |
| Methods | | | |
| Target population and subgroups | 4 | Describe characteristics of the base case population and subgroups analysed, including why they were chosen. | Page 4, Lines 147 to 152 |
| Setting and location | 5 | State relevant aspects of the system(s) in which the decision(s) need(s) to be made. | Page 4, Line 149 |
| Study perspective | 6 | Describe the perspective of the study and relate this to the costs being evaluated. | Page 2, Line 88 and page 3, lines 89 to 93 |
| Comparators | 7 | Describe the interventions or strategies being compared and state why they were chosen. | Page 2, lines 77 to 82 |
| Time horizon | 8 | State the time horizon(s) over which costs and consequences are being evaluated and say why appropriate. | Page 5 , lines 188 to 189 |
| Discount rate | 9 | Report the choice of discount rate(s) used for costs and outcomes and say why appropriate. | Page 5, lines 189 to 191 |
| Choice of health outcomes | 10 | Describe what outcomes were used as the measure(s) of benefit in the evaluation and their relevance for the type of analysis performed. | Page 5, lines 195 to 196 |
| Measurement of effectiveness | 11a | *Single study-based estimates:*Describe fully the design features of the single effectiveness study and why the single study was a sufficient source of clinical effectiveness data. | Page 3 , lines 95 to 101 |
|  | 11b | *Synthesis-based estimates*: Describe fully the methods used for identification of included studies and synthesis of clinical effectiveness data. | not applicable |
| Measurement and valuation of preference based outcomes | 12 | If applicable, describe the population and methods used to elicit preferences for outcomes. | Page 5 , lines 187 to 207 |
| Estimating resources and costs | 13a | *Single study-based economic evaluation:* Describe approaches used to estimate resource use associated with the alternative interventions. Describe primary or secondary research methods for valuing each resource item in terms of its unit cost. Describe any adjustments made to approximate to opportunity costs. | Page 4 , Lines 161 to 170 and page 5 line 171 to 172 |
|  | 13b | *Model-based economic evaluation:*Describe approaches and data sources used to estimate resource use associated with model health states. Describe primary or secondary research methods for valuing each resource item in terms of its unit cost. Describe any adjustments made to approximate to opportunity costs. | Not applicable |
| Currency, price date, and conversion | 14 | Report the dates of the estimated resource quantities and unit costs. Describe methods for adjusting estimated unit costs to the year of reported costs if necessary. Describe methods for converting costs into a common currency base and the exchange rate. | Page 5, lines 183 to 185 |
| Choice of model | 15 | Describe and give reasons for the specific type of decision-analytical model used. Providing a figure to show model structure is strongly recommended. | Page 4, lines 142 to 159 and supplementary Figure 1 |
| Assumptions | 16 | Describe all structural or other assumptions underpinning the decision-analytical model. | Page 2, lines 79 to 82, Page 3 lines 97 to 100, and Page 4 lines 165 to 168 |
| Analytical methods | 17 | Describe all analytical methods supporting the evaluation. This could include methods for dealing with skewed, missing, or censored data; extrapolation methods; methods for pooling data; approaches to validate or make adjustments (such as half cycle corrections) to a model; and methods for handling population heterogeneity and uncertainty. | Page 3 lines 107 to 110 |
| Results | | | |
| Study parameters | 18 | Report the values, ranges, references, and, if used, probability distributions for all parameters. Report reasons or sources for distributions used to represent uncertainty where appropriate. Providing a table to show the input values is strongly recommended. | Tables S5 and S6 in supplementary material and Table 2 |
| Incremental costs and outcomes | 19 | For each intervention, report mean values for the main categories of estimated costs and outcomes of interest, as well as mean differences between the comparator groups. If applicable, report incremental cost-effectiveness ratios. | Page 6 lines 217 to 231 and Page 9 lines 236 to 262, Table 4 and Table 5 |
| Characterizing uncertainty | 20a | *Single study-based economic evaluation:* Describe the effects of sampling uncertainty for the estimated incremental cost and incremental effectiveness parameters, together with the impact of methodological assumptions (such as discount rate, study perspective). | Not applicable |
|  | 20b | *Model-based economic evaluation:*Describe the effects on the results of uncertainty for all input parameters, and uncertainty related to the structure of the model and assumptions. | Page 5 lines 190 to 196 and Table 2 |
| Characterizing heterogeneity | 21 | If applicable, report differences in costs, outcomes, or cost-effectiveness that can be explained by variations between subgroups of patients with different baseline characteristics or other observed variability in effects that are not reducible by more information. | Not applicable |
| Discussion | | | |
| Study findings, limitations, generalisability, and current knowledge | 22 | Summarise key study findings and describe how they support the conclusions reached. Discuss limitations and the generalisability of the findings and how the findings fit with current knowledge. | Page 12 lines 289 to 317, Page 13 lines 335 to 369 |
| Other | | | |
| Source of funding | 23 | Describe how the study was funded and the role of the funder in the identification, design, conduct, and reporting of the analysis. Describe other non-monetary sources of support. | Page 14 lines 410 to 416 |
| Conflicts of interest | 24 | Describe any potential for conflict of interest of study contributors in accordance with journal policy. In the absence of a journal policy, we recommend authors comply with International Committee of Medical Journal Editors recommendations. | Page 15 lines 420 to 424 |

Supplementary Table S2. Paired T-test of energy content between HSR products in 2013 and 2016

| Variable | Variable 1 (2013 kJ per 100g) | Variable 2 (2016 kJ per 100g) |
| --- | --- | --- |
| Mean (x̄) | 1150 | 1143 |
| Variance (σ^2^) | 559721 | 564765 |
| Observations (n) | 1004 | 1004 |
|  |  |  |
| Pearson correlation coefficient (r) | 0.988 |  |
| Hypothesized mean difference (μ_0_) | 0 |  |
| Degrees of freedom (df) | 1003 |  |
| t stat | 1.980 |  |
| P(T ≤ t) one-tail | 0.024 |  |
| t critical one-tail | 1.646 |  |
| P(T≤ t) two-tail | 0.048 |  |
| t critical two-tail | 1.960 |  |

We conducted a paired two sample t-test in Excel using products available in 2013 that implemented the HSR system in 2016. As shown in the table above, we found a significant difference between the two samples at the 5% level of significance (i.e., t stat 1.98 > t critical 1.96).

# Table S3 .Total number of food products included by food categories and use of HSR labelling

| Food group name | Status | Number of food products avaiable in 2013 and 2016 | Number of food products available in 2013 with HSR in 2016 | Number of food products available in 2013 without HSR in 2016 | HSR uptake  (% of total) |
| --- | --- | --- | --- | --- | --- |
| Bread and bakery products | Included | 1990 | 153 | 1837 | 7.7 |
| Cereal and grain products | Included | 1664 | 230 | 1434 | 13.8 |
| Confectionery | Included | 1068 | 56 | 1012 | 5.2 |
| Convenience foods | Included | 979 | 84 | 895 | 8.6 |
| Dairy | Included | 1742 | 98 | 1644 | 5.6 |
| Edible oils and oil emulsions | Included | 266 | 16 | 250 | 6.0 |
| Eggs | Excluded | 33 |  | 33 | 3.4 |
| Fish and fish products | Included | 552 | 19 | 533 | 6.2 |
| Fruit and vegetables | Included | 2027 | 125 | 1902 | 7.4 |
| Meat and meat products | Included | 995 | 74 | 921 | 5.5 |
| Non-alcoholic beverages | Included | 1211 | 66 | 1145 | 3.2 |
| Sauces, dressings, spreads and dips | Included | 1791 | 58 | 1733 | 4.4 |
| Snack foods | Included | 409 | 18 | 391 | 2.7 |
| Sugars, honey and related products | Included | 259 | 7 | 252 | 7.7 |
| Special foods (e.g., baby food) | Excluded | n/a | n/a | n/a | n/a |
| Unable to be categorised | Excluded | n/a | n/a | n/a | n/a |
| Vitamins and supplements | Excluded | n/a | n/a | n/a | n/a |
| Alcohol | Excluded | n/a | n/a | n/a | n/a |
| **Total** | **13** | **14986** | **1004** | **13982** | **6.7%** |

# Table S4 Consumption weights of food categories for total daily energy intake for males

| **Age** | **Bread and bakery products** | **Cereal and grain products** | **Confectionery** | **Convenience foods** | **Dairy** | **Edible oils and oil emulsions** | **Fish and fish products** | **Fruit and vegetables** | **Meat and meat products** | **Non-alcoholic beverages** | **Sauces, dressings, spreads and dips** | **Snack foods** | **Sugars, honey and related products** |
| --- | --- | --- | --- | --- | --- | --- | --- | --- | --- | --- | --- | --- | --- |
| **2** | 0.078 | 0.088 | 0.075 | 0.182 | 0.085 | 0.020 | 0.170 | 0.034 | 0.132 | 0.023 | 0.016 | 0.067 | 0.030 |
| **3** | 0.097 | 0.083 | 0.061 | 0.161 | 0.082 | 0.019 | 0.179 | 0.041 | 0.130 | 0.020 | 0.012 | 0.077 | 0.037 |
| **4** | 0.112 | 0.102 | 0.048 | 0.175 | 0.079 | 0.025 | 0.123 | 0.042 | 0.099 | 0.024 | 0.012 | 0.116 | 0.044 |
| **5** | 0.119 | 0.088 | 0.048 | 0.183 | 0.082 | 0.028 | 0.092 | 0.039 | 0.130 | 0.020 | 0.024 | 0.121 | 0.026 |
| **6** | 0.118 | 0.093 | 0.080 | 0.180 | 0.078 | 0.029 | 0.086 | 0.037 | 0.143 | 0.020 | 0.021 | 0.073 | 0.041 |
| **7** | 0.102 | 0.093 | 0.052 | 0.187 | 0.080 | 0.020 | 0.081 | 0.037 | 0.148 | 0.021 | 0.037 | 0.119 | 0.025 |
| **8** | 0.097 | 0.108 | 0.043 | 0.179 | 0.063 | 0.021 | 0.099 | 0.037 | 0.218 | 0.017 | 0.016 | 0.075 | 0.028 |
| **9** | 0.110 | 0.101 | 0.049 | 0.199 | 0.070 | 0.022 | 0.077 | 0.047 | 0.144 | 0.025 | 0.021 | 0.102 | 0.032 |
| **10** | 0.091 | 0.092 | 0.112 | 0.175 | 0.073 | 0.018 | 0.085 | 0.037 | 0.135 | 0.023 | 0.018 | 0.109 | 0.033 |
| **11** | 0.100 | 0.111 | 0.058 | 0.172 | 0.068 | 0.018 | 0.111 | 0.045 | 0.174 | 0.020 | 0.016 | 0.083 | 0.023 |
| **12** | 0.101 | 0.106 | 0.059 | 0.212 | 0.071 | 0.022 | 0.099 | 0.035 | 0.150 | 0.023 | 0.017 | 0.079 | 0.026 |
| **13** | 0.083 | 0.102 | 0.148 | 0.173 | 0.058 | 0.022 | 0.071 | 0.027 | 0.147 | 0.026 | 0.021 | 0.099 | 0.024 |
| **14** | 0.106 | 0.087 | 0.059 | 0.196 | 0.068 | 0.029 | 0.053 | 0.032 | 0.179 | 0.027 | 0.015 | 0.126 | 0.023 |
| **15** | 0.089 | 0.126 | 0.036 | 0.172 | 0.066 | 0.020 | 0.128 | 0.032 | 0.137 | 0.028 | 0.025 | 0.117 | 0.025 |
| **16** | 0.114 | 0.097 | 0.071 | 0.198 | 0.066 | 0.023 | 0.078 | 0.025 | 0.158 | 0.030 | 0.015 | 0.102 | 0.023 |
| **17** | 0.108 | 0.109 | 0.091 | 0.175 | 0.063 | 0.030 | 0.068 | 0.034 | 0.162 | 0.029 | 0.018 | 0.095 | 0.019 |
| **18** | 0.089 | 0.180 | 0.045 | 0.148 | 0.050 | 0.020 | 0.075 | 0.044 | 0.168 | 0.023 | 0.014 | 0.127 | 0.017 |
| **19** | 0.091 | 0.131 | 0.086 | 0.151 | 0.080 | 0.042 | 0.087 | 0.030 | 0.157 | 0.020 | 0.020 | 0.067 | 0.037 |
| **20-24** | 0.110 | 0.116 | 0.051 | 0.180 | 0.065 | 0.027 | 0.103 | 0.031 | 0.120 | 0.024 | 0.029 | 0.123 | 0.020 |
| **25-29** | 0.086 | 0.121 | 0.058 | 0.185 | 0.062 | 0.024 | 0.105 | 0.028 | 0.164 | 0.023 | 0.021 | 0.108 | 0.016 |
| **30-34** | 0.091 | 0.115 | 0.084 | 0.171 | 0.054 | 0.024 | 0.078 | 0.038 | 0.153 | 0.021 | 0.030 | 0.119 | 0.021 |
| **35-39** | 0.102 | 0.111 | 0.067 | 0.184 | 0.046 | 0.028 | 0.121 | 0.032 | 0.133 | 0.020 | 0.021 | 0.119 | 0.016 |
| **40-44** | 0.097 | 0.116 | 0.079 | 0.172 | 0.046 | 0.028 | 0.110 | 0.039 | 0.160 | 0.018 | 0.029 | 0.087 | 0.019 |
| **45-49** | 0.103 | 0.120 | 0.073 | 0.174 | 0.042 | 0.032 | 0.117 | 0.035 | 0.141 | 0.018 | 0.019 | 0.106 | 0.022 |
| **50-54** | 0.097 | 0.114 | 0.087 | 0.163 | 0.045 | 0.028 | 0.103 | 0.043 | 0.141 | 0.015 | 0.020 | 0.127 | 0.018 |
| **55-59** | 0.097 | 0.113 | 0.108 | 0.153 | 0.039 | 0.025 | 0.126 | 0.037 | 0.167 | 0.012 | 0.020 | 0.088 | 0.016 |
| **60-64** | 0.098 | 0.132 | 0.075 | 0.142 | 0.043 | 0.034 | 0.121 | 0.038 | 0.172 | 0.011 | 0.029 | 0.089 | 0.016 |
| **65-69** | 0.095 | 0.118 | 0.054 | 0.141 | 0.039 | 0.034 | 0.152 | 0.035 | 0.171 | 0.011 | 0.023 | 0.110 | 0.018 |
| **70-74** | 0.103 | 0.121 | 0.065 | 0.151 | 0.045 | 0.031 | 0.163 | 0.041 | 0.155 | 0.009 | 0.019 | 0.079 | 0.018 |
| **75-79** | 0.101 | 0.101 | 0.066 | 0.128 | 0.042 | 0.028 | 0.127 | 0.036 | 0.166 | 0.011 | 0.019 | 0.158 | 0.016 |
| **80+** | 0.099 | 0.102 | 0.071 | 0.138 | 0.046 | 0.028 | 0.157 | 0.038 | 0.169 | 0.010 | 0.027 | 0.093 | 0.022 |

# Table S5. Consumption weights for food categories on total daily energy intake for females

| **Age** | **Bread and bakery products** | **Cereal and grain products** | **Confectionery** | **Convenience foods** | **Dairy** | **Edible oils and oil emulsions** | **Fish and fish products** | **Fruit and vegetables** | **Meat and meat products** | **Non-alcoholic beverages** | **Sauces, dressings, spreads and dips** | **Snack foods** | **Sugars, honey and related products** |
| --- | --- | --- | --- | --- | --- | --- | --- | --- | --- | --- | --- | --- | --- |
| **2** | 0.091 | 0.083 | 0.065 | 0.187 | 0.085 | 0.023 | 0.119 | 0.044 | 0.124 | 0.019 | 0.013 | 0.124 | 0.022 |
| **3** | 0.113 | 0.088 | 0.067 | 0.139 | 0.081 | 0.025 | 0.058 | 0.035 | 0.216 | 0.020 | 0.015 | 0.108 | 0.034 |
| **4** | 0.086 | 0.095 | 0.051 | 0.186 | 0.077 | 0.020 | 0.124 | 0.043 | 0.175 | 0.016 | 0.020 | 0.080 | 0.027 |
| **5** | 0.102 | 0.099 | 0.066 | 0.177 | 0.073 | 0.025 | 0.077 | 0.041 | 0.159 | 0.022 | 0.014 | 0.113 | 0.032 |
| **6** | 0.100 | 0.125 | 0.061 | 0.149 | 0.070 | 0.044 | 0.100 | 0.042 | 0.136 | 0.021 | 0.015 | 0.095 | 0.042 |
| **7** | 0.105 | 0.096 | 0.060 | 0.168 | 0.075 | 0.028 | 0.142 | 0.047 | 0.141 | 0.018 | 0.013 | 0.086 | 0.021 |
| **8** | 0.117 | 0.119 | 0.075 | 0.173 | 0.085 | 0.024 | 0.095 | 0.046 | 0.127 | 0.021 | 0.011 | 0.079 | 0.026 |
| **9** | 0.105 | 0.123 | 0.085 | 0.185 | 0.078 | 0.028 | 0.079 | 0.036 | 0.144 | 0.019 | 0.015 | 0.077 | 0.028 |
| **10** | 0.107 | 0.094 | 0.050 | 0.204 | 0.077 | 0.025 | 0.092 | 0.046 | 0.123 | 0.019 | 0.022 | 0.112 | 0.030 |
| **11** | 0.099 | 0.129 | 0.080 | 0.160 | 0.068 | 0.022 | 0.119 | 0.041 | 0.118 | 0.023 | 0.031 | 0.089 | 0.021 |
| **12** | 0.088 | 0.112 | 0.080 | 0.176 | 0.088 | 0.031 | 0.082 | 0.033 | 0.139 | 0.025 | 0.016 | 0.093 | 0.037 |
| **13** | 0.112 | 0.121 | 0.070 | 0.156 | 0.089 | 0.022 | 0.065 | 0.031 | 0.127 | 0.026 | 0.019 | 0.138 | 0.023 |
| **14** | 0.103 | 0.101 | 0.064 | 0.158 | 0.076 | 0.032 | 0.113 | 0.039 | 0.102 | 0.028 | 0.023 | 0.106 | 0.054 |
| **15** | 0.133 | 0.105 | 0.075 | 0.178 | 0.085 | 0.024 | 0.078 | 0.033 | 0.112 | 0.026 | 0.030 | 0.093 | 0.029 |
| **16** | 0.098 | 0.128 | 0.075 | 0.151 | 0.080 | 0.017 | 0.059 | 0.035 | 0.136 | 0.016 | 0.016 | 0.164 | 0.025 |
| **17** | 0.118 | 0.107 | 0.095 | 0.167 | 0.061 | 0.018 | 0.104 | 0.031 | 0.108 | 0.023 | 0.018 | 0.118 | 0.033 |
| **18** | 0.068 | 0.132 | 0.112 | 0.205 | 0.061 | 0.016 | 0.034 | 0.038 | 0.125 | 0.019 | 0.030 | 0.135 | 0.024 |
| **19** | 0.109 | 0.104 | 0.076 | 0.178 | 0.051 | 0.018 | 0.091 | 0.043 | 0.115 | 0.019 | 0.023 | 0.163 | 0.012 |
| **20-24** | 0.099 | 0.106 | 0.085 | 0.187 | 0.059 | 0.036 | 0.061 | 0.033 | 0.159 | 0.021 | 0.022 | 0.112 | 0.021 |
| **25-29** | 0.094 | 0.109 | 0.083 | 0.178 | 0.050 | 0.024 | 0.108 | 0.030 | 0.145 | 0.017 | 0.026 | 0.119 | 0.017 |
| **30-34** | 0.093 | 0.121 | 0.076 | 0.162 | 0.045 | 0.024 | 0.120 | 0.034 | 0.146 | 0.016 | 0.032 | 0.113 | 0.018 |
| **35-39** | 0.106 | 0.118 | 0.075 | 0.174 | 0.048 | 0.029 | 0.102 | 0.036 | 0.147 | 0.017 | 0.024 | 0.103 | 0.021 |
| **40-44** | 0.096 | 0.116 | 0.087 | 0.159 | 0.047 | 0.026 | 0.109 | 0.037 | 0.158 | 0.015 | 0.025 | 0.109 | 0.016 |
| **45-49** | 0.106 | 0.122 | 0.071 | 0.143 | 0.042 | 0.034 | 0.125 | 0.038 | 0.174 | 0.015 | 0.031 | 0.085 | 0.015 |
| **50-54** | 0.106 | 0.113 | 0.096 | 0.156 | 0.035 | 0.030 | 0.118 | 0.041 | 0.147 | 0.011 | 0.028 | 0.100 | 0.017 |
| **55-59** | 0.096 | 0.122 | 0.080 | 0.167 | 0.038 | 0.026 | 0.125 | 0.040 | 0.148 | 0.011 | 0.024 | 0.106 | 0.017 |
| **60-64** | 0.099 | 0.121 | 0.081 | 0.147 | 0.038 | 0.032 | 0.114 | 0.039 | 0.160 | 0.010 | 0.027 | 0.116 | 0.017 |
| **65-69** | 0.097 | 0.113 | 0.072 | 0.161 | 0.043 | 0.032 | 0.146 | 0.036 | 0.170 | 0.010 | 0.035 | 0.070 | 0.016 |
| **70-74** | 0.093 | 0.106 | 0.074 | 0.150 | 0.044 | 0.029 | 0.137 | 0.038 | 0.162 | 0.012 | 0.038 | 0.103 | 0.016 |
| **75-79** | 0.098 | 0.094 | 0.053 | 0.138 | 0.044 | 0.030 | 0.141 | 0.043 | 0.174 | 0.009 | 0.025 | 0.128 | 0.023 |
| **80+** | 0.105 | 0.104 | 0.079 | 0.132 | 0.044 | 0.032 | 0.106 | 0.039 | 0.203 | 0.012 | 0.030 | 0.095 | 0.019 |

# Table S6. Composition of food products displaying HSR in 2016 and composition of the same products in 2013 ( n=1009)

| **Food group included** | **Average energy (kJ per 100g)** | | | **Mean difference** | **% difference** |
| --- | --- | --- | --- | --- | --- |
|  | **2013** | **2016** | |  |  |
| Bread and bakery products | 1585 | | 1581 | -3.3 | -0.2 |
| Cereal and grain products | 1521 | | 1513 | -7.9 | -0.5 |
| Confectionery | 2070 | | 2089 | 19.7 | 1.0 |
| Convenience foods | 444 | | 433 | -10.9 | -2.5 |
| Dairy | 608 | | 594 | -13.4 | -2.2 |
| Edible oils and oil emulsions | 2724 | | 2706 | -18.1 | -0.7 |
| Fish and fish products | 721 | | 720 | -1.0 | -0.1 |
| Fruit and vegetables | 881 | | 881 | -0.6 | -0.1 |
| Meat and meat products | 828 | | 824 | -4.1 | -0.5 |
| Non-alcoholic beverages | 213 | | 208 | -4.6 | -2.1 |
| Sauces, dressings, spreads and dips | 1046 | | 981 | -64.7 | -6.2 |
| Snack foods | 2013 | | 2079 | 65.8 | 3.3 |
| Sugars, honey and related products | 1454 | | 1435 | -19.7 | -1.4 |
| **Total average (p value)** | **1150** | | **1143** | **-7.1 (0.04)** | **-0.6** |

Abbreviations: kJ: kilojoules

# Table S7. Intervention effect size for males by age

|  | **Voluntary scenario** | | | **Mandatory scenario** | | |
| --- | --- | --- | --- | --- | --- | --- |
| Age | Average change in energy (kJ per day) | Average change in kilograms (kg) | Average change in BMI | Average change in energy (kJ per day) | Average change in kilograms (kg) | Average change in BMI |
| 2 | -1.0682 | -0.004 | -0.005 | -15.249 | -0.058 | -0.068 |
| 3 | -0.8514 | -0.003 | -0.003 | -12.482 | -0.049 | -0.048 |
| 4 | -0.9273 | -0.004 | -0.003 | -12.363 | -0.051 | -0.044 |
| 5 | -0.8833 | -0.004 | -0.003 | -11.147 | -0.048 | -0.038 |
| 6 | -1.1086 | -0.005 | -0.004 | -15.635 | -0.071 | -0.049 |
| 7 | -1.2608 | -0.006 | -0.004 | -16.469 | -0.078 | -0.048 |
| 8 | -1.2254 | -0.006 | -0.003 | -16.472 | -0.082 | -0.047 |
| 9 | -1.2977 | -0.007 | -0.004 | -17.044 | -0.090 | -0.048 |
| 10 | -1.0759 | -0.006 | -0.003 | -13.723 | -0.076 | -0.038 |
| 11 | -1.0438 | -0.006 | -0.003 | -13.914 | -0.082 | -0.038 |
| 12 | -1.6550 | -0.010 | -0.004 | -21.617 | -0.136 | -0.055 |
| 13 | -1.1300 | -0.008 | -0.003 | -13.771 | -0.093 | -0.035 |
| 14 | -1.3691 | -0.010 | -0.003 | -16.209 | -0.117 | -0.041 |
| 15 | -1.1106 | -0.009 | -0.003 | -13.326 | -0.104 | -0.036 |
| 16 | -1.3936 | -0.012 | -0.004 | -17.286 | -0.148 | -0.048 |
| 17 | -1.3968 | -0.013 | -0.004 | -18.114 | -0.170 | -0.053 |
| 18 | -0.7314 | -0.008 | -0.002 | -7.100 | -0.074 | -0.024 |
| 19 | -1.5075 | -0.015 | -0.005 | -22.317 | -0.223 | -0.069 |
| 20-24 | -1.3191 | -0.013 | -0.004 | -16.285 | -0.163 | -0.051 |
| 25-29 | -1.4127 | -0.014 | -0.004 | -17.140 | -0.171 | -0.054 |
| 30-34 | -1.1452 | -0.011 | -0.004 | -13.052 | -0.131 | -0.042 |
| 35-39 | -1.1255 | -0.011 | -0.004 | -12.391 | -0.124 | -0.039 |
| 40-44 | -1.1664 | -0.012 | -0.004 | -14.944 | -0.149 | -0.047 |
| 45-49 | -1.0134 | -0.010 | -0.003 | -11.696 | -0.117 | -0.038 |
| 50-54 | -0.8807 | -0.009 | -0.003 | -8.274 | -0.083 | -0.027 |
| 55-59 | -0.9870 | -0.010 | -0.003 | -11.658 | -0.117 | -0.038 |
| 60-64 | -0.8645 | -0.009 | -0.003 | -10.866 | -0.109 | -0.036 |
| 65-69 | -0.8403 | -0.008 | -0.003 | -9.716 | -0.097 | -0.033 |
| 70-74 | -0.9107 | -0.009 | -0.003 | -11.334 | -0.113 | -0.038 |
| 75-79 | -0.5566 | -0.006 | -0.002 | -3.798 | -0.038 | -0.013 |
| 80+ | -0.7827 | -0.008 | -0.003 | -9.623 | -0.096 | -0.034 |

*Abbreviations: BMI: body mass index*

Table S8. Intervention effect size for females by age

|  | **Voluntary scenario** | | | **Mandatory scenario** | | |
| --- | --- | --- | --- | --- | --- | --- |
| Age | Average change in energy (kJ per g) | Average change in kilograms (kg) | Average change in BMI | Average change in energy (kJ per g) | Average change in kilograms (kg) | Average change in BMI |
| 2 | -0.758 | -0.003 | -0.004 | -8.941 | -0.039 | -0.047 |
| 3 | -0.729 | -0.003 | -0.003 | -9.711 | -0.044 | -0.045 |
| 4 | -0.928 | -0.004 | -0.004 | -12.467 | -0.058 | -0.051 |
| 5 | -0.897 | -0.004 | -0.003 | -11.487 | -0.056 | -0.044 |
| 6 | -0.742 | -0.004 | -0.003 | -10.599 | -0.054 | -0.039 |
| 7 | -0.842 | -0.005 | -0.003 | -11.670 | -0.063 | -0.041 |
| 8 | -0.856 | -0.005 | -0.003 | -12.346 | -0.070 | -0.041 |
| 9 | -1.126 | -0.007 | -0.004 | -15.198 | -0.091 | -0.048 |
| 10 | -1.137 | -0.007 | -0.003 | -14.486 | -0.092 | -0.044 |
| 11 | -1.039 | -0.007 | -0.003 | -14.392 | -0.097 | -0.043 |
| 12 | -1.215 | -0.009 | -0.004 | -17.384 | -0.124 | -0.051 |
| 13 | -0.866 | -0.007 | -0.003 | -10.333 | -0.079 | -0.031 |
| 14 | -1.011 | -0.008 | -0.003 | -13.728 | -0.113 | -0.043 |
| 15 | -1.148 | -0.010 | -0.004 | -16.561 | -0.148 | -0.056 |
| 16 | -0.372 | -0.004 | -0.001 | 0.088 | 0.001 | 0.000 |
| 17 | -0.806 | -0.009 | -0.003 | -8.925 | -0.095 | -0.035 |
| 18 | -1.022 | -0.012 | -0.004 | -10.469 | -0.124 | -0.045 |
| 19 | -1.032 | -0.010 | -0.004 | -9.651 | -0.097 | -0.035 |
| 20-24 | -1.079 | -0.011 | -0.004 | -12.407 | -0.124 | -0.046 |
| 25-29 | -0.997 | -0.010 | -0.004 | -10.736 | -0.107 | -0.040 |
| 30-34 | -0.873 | -0.009 | -0.003 | -10.272 | -0.103 | -0.038 |
| 35-39 | -0.989 | -0.010 | -0.004 | -11.729 | -0.117 | -0.044 |
| 40-44 | -0.839 | -0.008 | -0.003 | -9.929 | -0.099 | -0.037 |
| 45-49 | -0.808 | -0.008 | -0.003 | -10.648 | -0.106 | -0.041 |
| 50-54 | -0.809 | -0.008 | -0.003 | -9.462 | -0.095 | -0.036 |
| 55-59 | -0.864 | -0.009 | -0.003 | -10.060 | -0.101 | -0.039 |
| 60-64 | -0.658 | -0.007 | -0.003 | -6.765 | -0.068 | -0.027 |
| 65-69 | -0.963 | -0.010 | -0.004 | -12.933 | -0.129 | -0.050 |
| 70-74 | -0.787 | -0.008 | -0.003 | -9.675 | -0.097 | -0.038 |
| 75-79 | -0.621 | -0.006 | -0.003 | -6.833 | -0.068 | -0.028 |
| 80+ | -0.729 | -0.007 | -0.003 | -9.034 | -0.090 | -0.038 |

*Abbreviations: BMI: body mass index*
