# Supplementary figures and images for: Cost-Effectiveness of Product Reformulation in Response to the Health Star Rating Food Labelling System in Australia
Source: Nutrients. 2018 May 14;10(5):614. doi: 10.3390/nu10050614 (PMC5986494; doi:10.3390/nu10050614)

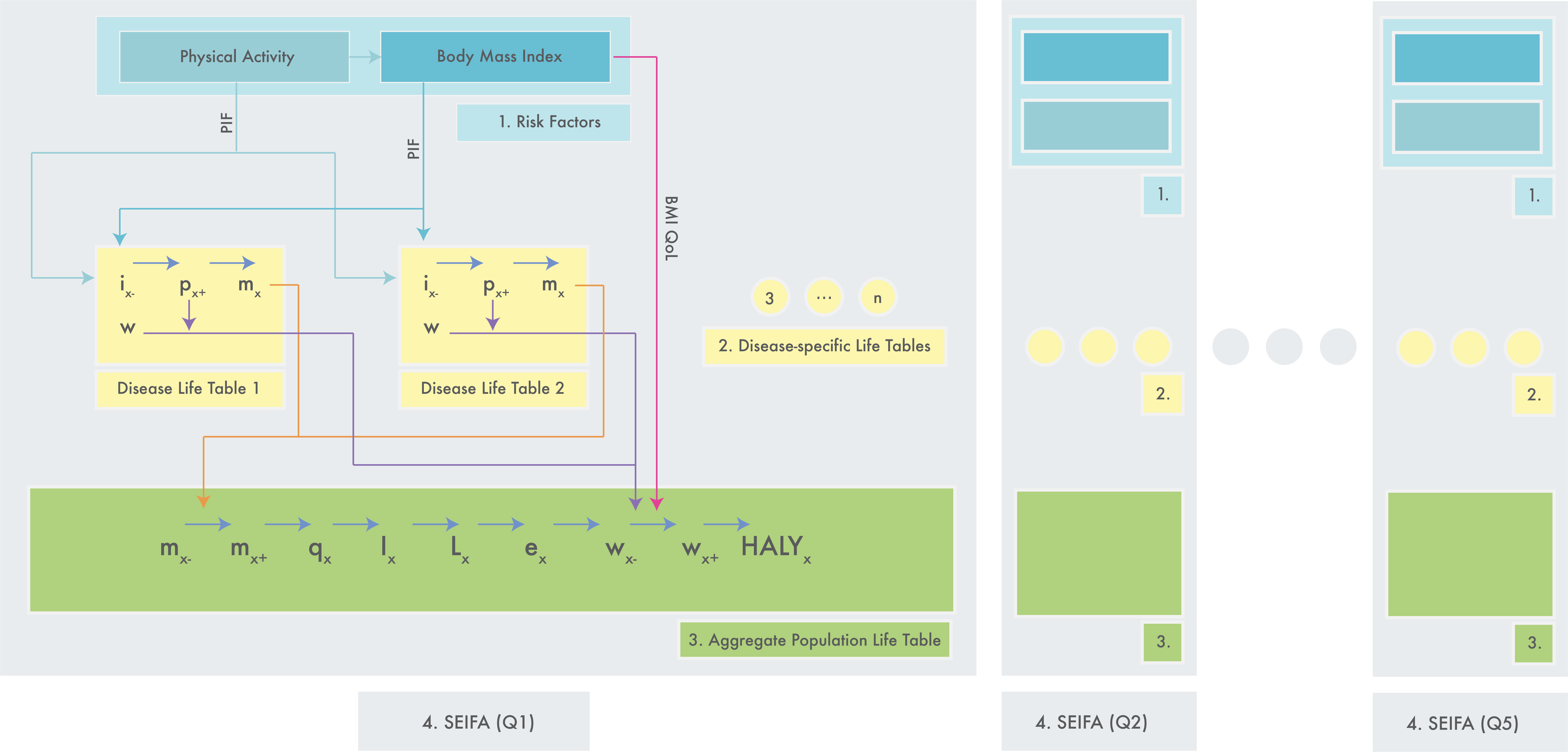

Supplement: Supplementary file 1 [file nutrients-10-00614-s001.zip › Supplementary Figure 1.png]
